# Retraction: MicroRNA-34a alleviates steroid-induced avascular necrosis of femoral head by targeting Tgif2 through OPG/RANK/RANKL signaling pathway

**DOI:** 10.3389/ebm.2024.10275

**Published:** 2024-06-25

**Authors:** EBM Editorial Office

Following publication, the authors contacted the Editorial Office to request the retraction of the cited article, stating that concerns were raised on the PubPeer platform regarding the reuse of certain images, as well as concerns over incomplete data. Particularly, in Figure 3, Panels c and d appear to overlap. Those panels also show duplication with Figure 11B from Zhang et al 2017 and Figure 5 from Yu et al 2019 (both retracted sources). Further, in Tables 2, 3 values are shown as means ± standard deviation and no exact measurements are provided. Therefore, the article has been retracted.

**FIGURE 3:**
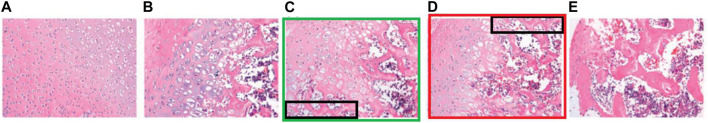
Comparison of the morphology changes in the femoral head by HE staining among (×100) among normal control, model control, negative control, miR-34a mimics and miR-34a inhibitor groups. **(A)**, normal control group; **(B)**, model control group; **(C)**, negative control group; **(D)**, miR-34a mimics group; **(E)**, miR-34a inhibitors group; miR-34a, miRNA-34a. (A color version of this figure is available in the online journal).

**TABLE 2:** Comparison of parameters of trabecular in the cancellous bone within the unit volume of the center of the femoral head between normal control group and model control group.

Group	BV/TV	BS/BV	Tb.Th	Tb.N
Normal control group	0.72 ± 0.07	25.07 ± 2.52	0.14 ± 0.02	6.34 ± 0.63
model control group	0.59 ± 0.06^a^	21.25 ± 2.10^a^	0.13 ± 0.01	5.83 ± 0.58^a^

^a^ Compared with the normal control group, *p* < 0.05; BV/TV, bone volume/total volume. BS/BV, bone surface area/bone volume; Tb.Th, trabecular thickness; Tb.N, trabecular number.

**TABLE 3:** Comparison of parameters of trabecular in the cancellous bone within the unit volume of the center of the femoral head among normal control, model control, negative control, miR-34a mimics and miR-34a inhibitor groups.

Group	BV/TV	BS/BV	Tb.Th	Tb.N
Normal control	0.71 ± 0.07	24.92 ± 2.51	0.14 ± 0.03	7.02 ± 0.69
Model control	0.55 ± 0.05^a^	20.13 ± 2.01^a^	0.13 ± 0.02	5.65 ± 0.56^a^
Negative control	0.52 + 0.05^a^	20.07 ± 2.00^a^	0.13 ± 0.02	5.63 ± 0.56^a^
miR-34a inhibitor	0.46 ± 0.04^a^ ^,^ ^b^	17.75 ± 1.75^a^ ^,^ ^b^	0.15 ± 0.04	5.00 ± 0.51^a^ ^,^ ^b^
miR-34a mimics	0.63 ± 0.06^a^ ^,^ ^b^	22.39 ± 2.22^a^ ^,^ ^b^	0.14 ± 0.02	6.39 + 0.63^a^ ^,^ ^b^

^a^ Compared to normal control group, *p* < 0.05.

^b^ Compared to model control group, *p* < 0.05. BV/TV, bone volume/total volume; BS/BV, bone surface area/bone volume; Tb.Th, trabecular thickness; Tb.N, trabecular number.

Figure 3 appears to show an overlap between panels (c) and (d).

Tables 2, 3 show values as means +/− deviation, no exact measurements provided.

This retraction was approved by the Editor-in-Chief of Experimental Biology and Medicine. The authors received communication regarding the retraction but did not respond. The communication has been recorded by the publisher.

